# Structural Activity and HAD Inhibition Efficiency of Pelargonidin and Its Glucoside—A Theoretical Approach

**DOI:** 10.3390/molecules27228016

**Published:** 2022-11-18

**Authors:** Rangasamy Praveena, Athinarayanan Balasankar, Kanakaraj Aruchamy, Taehwan Oh, Veerababu Polisetti, Subramaniyan Ramasundaram, Kandasamy Anbazhakan

**Affiliations:** 1 Department of Chemistry, Bannari Amman Institute of Technology, Sathyamangalam 638401, India; 2 Department of Physics, Gobi Arts & Science College, Gobichettipalayam 638453, India; 3 School of Chemical Engineering, Yeungnam University, Gyeongsan 38541, Republic of Korea; 4 Wallenberg Wood Science Center, Department of Fibre and Polymer Technology, School of Engineering Sciences in Chemistry, Biotechnology and Health, KTH Royal Institute of Technology, SE-100 44 Stockholm, Sweden

**Keywords:** anthocyanins, pelargonidin, density functional theory, structural activity, in silico, BOILED-egg analysis, molecular docking, MD simulations

## Abstract

Anthocyanins are an important pharmaceutical ingredient possessing diet regulatory, antioxidant, anticancer, antidiabetic, anti-obesity, antimicrobial, and anti-inflammatory properties. Pelargonidin is an important anthocyanin-based orange-red flavonoid compound used in drugs for treating hypoglycemia, retinopathy, skeletal myopathy, etc. The main sources of pelargonidin are strawberries and food products with red pigmentation. There is a lack of evidence for supporting its use as an independent supplement. In the present study, pelargonidin and pelargonidin-3-O-glucoside are studied for their structural properties using quantum chemical calculations based on density functional theory. The results confirmed that the parent compound and its glycosylated derivative acted as good electron donors. Electrostatic potential, frontier molecular orbitals, and molecular descriptor analyses also substantiated their electron donating properties. Furthermore, based on the probability, a target prediction was performed for pelargonidin and pelargonidin-3-O-glucoside. Hydroxyacyl-coenzyme A dehydrogenase was chosen as an enzymatic target of interest, since the presence work focuses on glucuronidated compounds and their efficacy over diabetes. Possible interactions between these compounds and a target with nominable binding energies were also evaluated. Further, the structural stability of these two compounds were also analyzed using a molecular dynamics simulation.

## 1. Introduction

Secondary metabolites derived from fruits, especially berries, are common in daily human diet. In particular, dark red and blue colored berry fruits contain high anthocyanins and glucosyl flavonoids content. These compounds protect human health against oxidative stress, cell damage, and cardiac difficulties [1,2,3]. Pelargonidin is an anthocyanins group of secondary metabolites having a hydroxyl group at the flavonoid skeleton’s C-7 position. The foods rich in 7-hydroxyflavonoids are radishes (*Raphanus sativus*), black raspberries (*Rubus occidentalis*), and strawberries (*Fragaria* × *ananassa*). Elderberries (*Sambucus*), sour cherries (*Prunus cerasus*), and red raspberries (*Rubus idaeus*) contain lower concentrations of 7-hydroxyflavonoids. The other food sources reported as containing pelargonidin include cereals, gigantic butterburs (*Petasites japonicus*), goji, cherry tomatoes (*Solanum lycopersicum* var. *cerasiforme*), and common corn salad (*Valerianella locusta*) [4,5,6,7,8]. 

Numerous cell culture studies were conducted for finding the effect of anthocyanins on cytokine production and other aspects of immune function [9,10,11,12]. However, the majority of these studies used the parent, unmetabolized anthocyanins, frequently at high doses, which may not be physiologically relevant [13,14,15,16,17,18,19]. Strawberries, a common fruit, are very high in anthocyanins, especially pelargonidin-3-O-glucoside (Pg-3-glc) [20]. Three pharmacokinetic investigations have identified glucuronidated pelargonidin as the main metabolite, but the exact role of glucuronidation is unclear, and as glucuronidated pelargonidin chemicals are not now commercially accessible, and they cannot be studied in cell culture models. Strawberries, a highly consumed fruit by different age groups all over the world contains polyphenolic compounds that are sodium free, cholesterol free, and supportive in low calorie diets prescribed for diabetic patients [21]. During the digestion process, the glycosyl flavonoids, for example, pelargonidin glycosides ((2S,3R,4S,5S,6R)-2-[5,7-dihydroxy-2-(4-hydroxyphenyl)chromenylium-3-yl]oxy-6-(hydroxymethyl)oxane-3,4,5-triol), get excreted from the body as result of absorption and metabolism. Investigations on absorption and metabolism of these anthocyanins revealed that after consumption, acetylated forms of pelargonidin glycosides were found in urine samples of humans and experimental animals [22].

Because of their antioxidant property, pelargonidin (2-(4-hydroxyphenyl)chromenylium-3,5,7-triol)—(C_15_H_11_O_5_^+^) and pelargonidin 3-O-𝛽-glucopyranoside or pelargonidin-3-O-glucoside—(C_21_H_21_O_10_^+^), have been used as a replacement for food additives such as alloxan, monosodium glutamate, aspartame, etc. These additives are known to pose serious threat to human health as they cause DNA damage, chromosomal aberration and diabetes [23]. Thus, investigation of inhibition potency over specific targets is crucial for exploring their use in a wide spectrum of pharmacological applications. Moreover, studies focusing their bioavailability and metabolism can be highly helpful for estimating their antioxidative capacity. The insights gained from the structural activities over a specified target can be useful to increase the understanding of the medicinal properties of pelargonidin and pelargonidin-3-O-glucoside. 

The present study has been framed to theoretically explore the structural properties and inhibition efficiency of pelargonidin and its glucoside pelargonidin-3-O glucoside over the specified target, hydroxyacyl-coenzyme A dehydrogenase. Structural optimization was performed using density functional theory. The Gaussian 09 program, Gauss view 05, and Chemcraft software (b622b_win64) were used for simulations. The Auto Dock program and Lamarckian genetic algorithm were used for molecular docking studies. The electron donating properties of pelargonidin and pelargonidin-3-O-glucoside were assessed using electrostatic potential, frontier molecular orbitals (FMO) and molecular descriptor analyses.

## 2. Computation Detail

**Figure 1:**
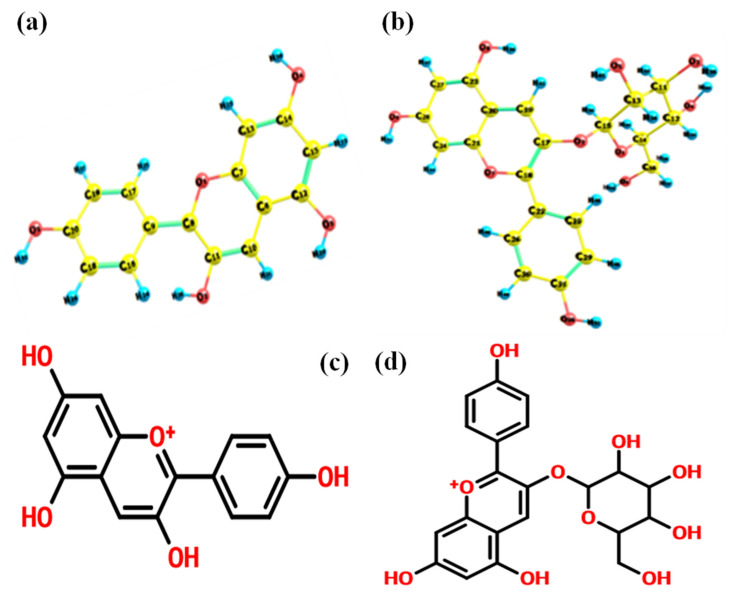
Optimized conformers of (**a**) pelargonidin, (**b**) pelargonidin-3-O-glucoside, (**c**) pelargonidin 2D conformer and (**d**) pelargonidin-3-O-glucoside 2D conformer.

In order to study the molecular properties of flavonoids, their ground state energies must be known. Optimization of structural features was performed using density functional theory. The initial geometries of the molecules are obtained from PUBCHEM-NCBI database and used for optimization. The structure of optimized conformers of pelargonidin and pelargonidin-3-O glucoside are shown in Figure 1a–d. The triple zeta valences basis set 6-311G(d,p), along with the exchange correlation functional B3LYP has been adopted here, since the considered molecules are highly delocalized. The simulations are supported by the Gaussian 09 program [24]. The visualization of the computed properties was made through Gauss view 05 and Chemcraft software. Molecular target prediction and BOILED-Egg analysis were assisted by SwissADME server. Docking analysis was supported by Auto Dock using Lamarckian genetic algorithm and the visualizations were made using UCF Chimera molecular visualizer. Corresponding molecular dynamics (MD) simulations were performed using GROMACS (2022.3) software [25].

## 3. Discussion

### 3.1. Frontier Molecular Orbital (FMO) Analysis

**Figure 2:**
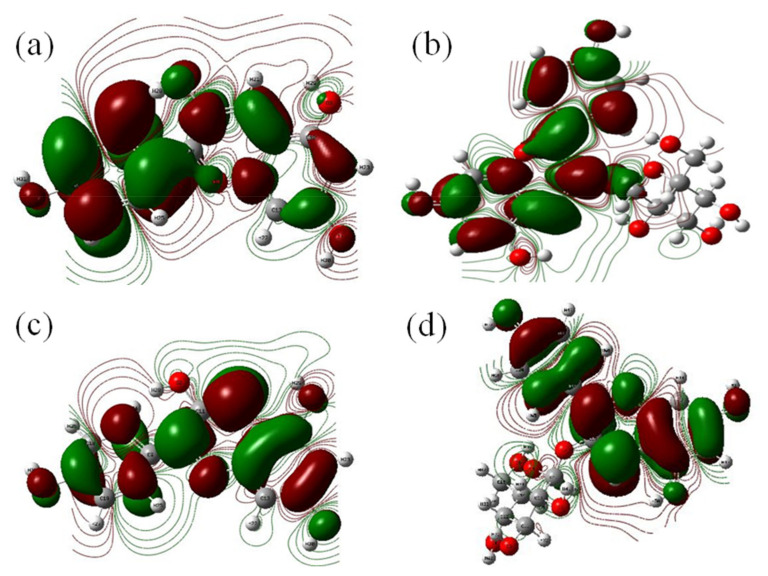
Lowest unoccupied molecular orbitals (LUMO) and highest occupied molecular orbitals (HOMO) derived from frontier molecular orbitals (FMO) analysis: (**a**) LUMO of pelargonidin; (**b**) LUMO of pelargonidin-3-O-glucoside; (**c**) HOMO of pelargonid; and (**d**) HUMO of pelargonidin-3-O-glucoside.

The electrophilic and nucleophilic properties of the molecular systems through interaction between the occupied and unoccupied molecular orbitals or energy levels are analyzed with the help of FMO models [26]. Figure 2a–d shows the FMO models of pelargonidin and pelargonidin-3-O-glucoside. In pelargonidin, it is observed that electron accepting л* type orbitals, otherwise known as lowest unoccupied molecular orbitals (LUMO), are spread over the A and C rings of pelargonidin. The electron donating л type orbitals donors or highest occupied molecular orbital (HOMO) are found to spread over the whole molecular surface and majorly over the B-ring which is the chief reactive site or donating site in the case of flavonoids. This observation clearly confirms the electron donating role of hydroxyl units and carbonyl carbon units of the B-ring in pelargonidin. Similarly, in pelargonidin-3-O-glucoside, л* orbitals (LUMO) are spread over the whole molecule and л orbitals (HOMO) are majorly concentrated over carbonyl carbon units present in the C ring, and hydroxyl units present in the B-ring, respectively. In both the compounds, the B-ring seems to be a prominent electron donor. The energy gap between occupied and unoccupied orbitals of pelargonidin was found to be 1.85 eV, for pelargonidin-3-O-glucoside, the energy gap was 1.91 eV. Observed energy gap values seem to support pelargonidin in terms of reactivity. Based on the FMO observations the structural activities of the two flavonoids are found to be in the order: pelargonidin > pelargonidin-3-O-glucoside.

### 3.2. Molecular Electrostatic Potential (MEP) Analysis 

**Figure 3:**
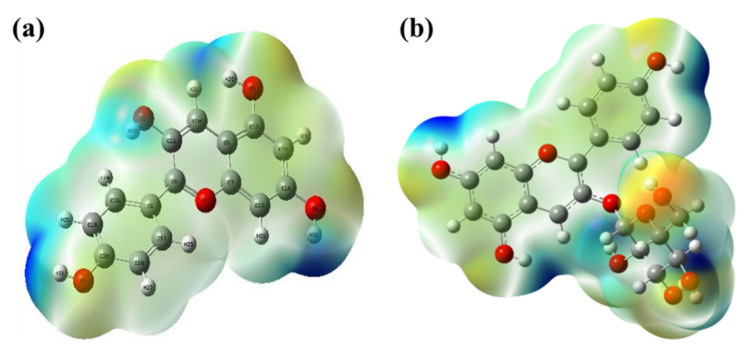
Molecular electrostatic potential (MEP) diagrams: (**a**) pelargonidin; and (**b**) pelargonidin-3-O-glucoside.

The chemical reactivity of pelargonidin and pelargonidin-3-O-glucoside can be well understood with the help of surface electrostatic potential [27]. From Figure 3a,b. the following observations were made. For both of these flavonoids, the highest electrostatic potential regions are observed over the hydroxyl units present in the A, B and C rings, which is an indication of their readiness to scavenge the free radicals, whereas heteroatom (oxygen) had the lowest electrostatic potential energy due to its valence electrons. Furthermore, the glycoside unit seems to exhibit lower electrostatic energy regions. Based on these observations, the chemical reactivity of pelargonidin was validated to be superior to pelargonidin-3-O-glucoside.

### 3.3. Molecular Descriptive Parameters

The molecular descriptive parameters estimated for pelargonidin and pelargonidin-3-O-glucoside are provided in Table 1. The difference between the ionization potential (IP) of pelargonidin and pelargonidin-3-O-glucoside was 0.12 eV, so both the compounds require same amount of energy to remove an electron from their structure. The magnitude of electron affinity (EA) of these compounds seems low. The lower EA makes these compounds poor electron acceptors. The EA of these compound differed by 0.17 eV. The hardness and softness values obtained for these compounds indicated their flexibility towards reactions. Electronegativity and electrophilicity values with minor order of energy difference are in line with above observations, confirming them to be better electron donor than electron acceptors [28].

### 3.4. Insilico Analysis

#### 3.4.1. BOILED-Egg Analysis

**Figure 4:**
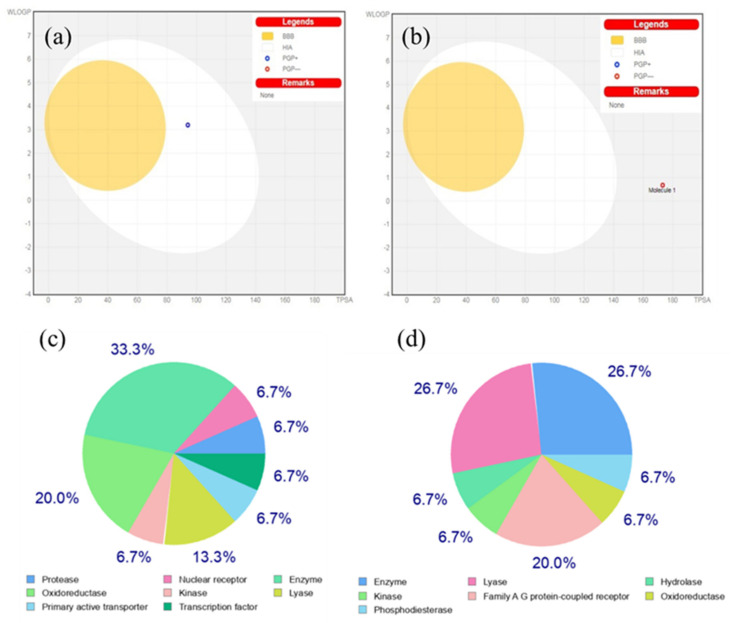
BOILED−egg graphs: (**a**) pelargonidin; and (**b**) pelargonidin−3−O−glucoside. Target prediction report: (**c**) pelargonidin; and (**d**) pelargonidin-3-O-glucoside.

The Brain Or IntestinaL Estimated permeation technique (BOILED-Egg) is a precise prediction model that operates by calculating the polarity and lipophilicity of tiny compounds. Due to the model’s speed, accuracy, conceptual simplicity, and clear graphical output, concurrent predictions for brain and intestine permeation are produced from the same two physicochemical descriptors and directly translated into molecular design. The BOILED-Egg may be used in a range of contexts, from the screening of chemical libraries during the preliminary stages of drug discovery to the assessment of potential therapeutic candidates [29]. From the Figure 4a,b, the white region is the physicochemical space of molecules with highest probability of being absorbed by the gastrointestinal tract, and the yellow region (yolk) is the physicochemical space of molecules with highest probability to permeate to the brain. Yolk and white areas are not mutually exclusive. Human intestinal absorption (HIA) and access to the blood–brain barrier (BBB) concentrations of these compounds were predicted using their cLogP and TPSA values. (Figure 4a,b). The presence of pelargonidin in the white region of the plot suggests that this molecule can be absorbed by the human intestine, whereas, pelargonidin-3-O-glucoside present in grey region of the plot suggests that this molecule penetrates neither HIA nor BBB. Additionally, this boiled-egg model predicted whether pelargonidin and its glucoside, and its derivatives were P-glycoprotein substrates (PGP+) and PGP-. While blue dots (PGP+) reflect the substances that are projected to pass into the CNS and are substrates of PGP, red dots (PGP-) represent substances that are not substrates of the PGP CNS efflux transporter.

#### 3.4.2. Molecular Target Prediction and Docking Analysis

**Figure 5:**
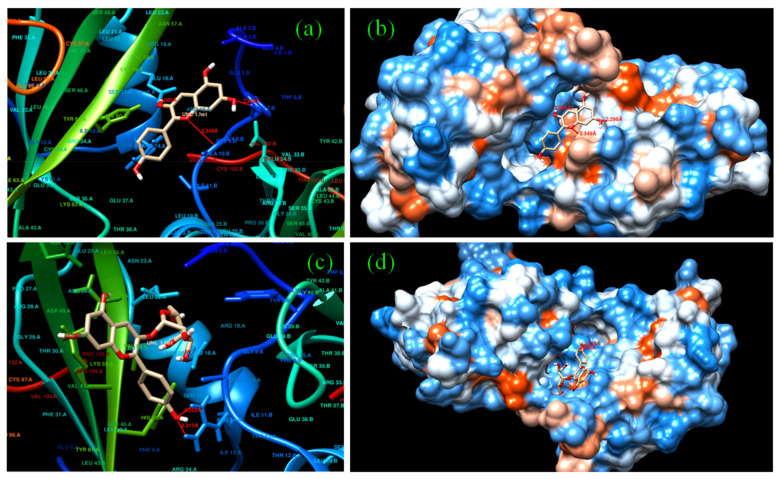
Docked poses of (**a**,**b**) pelargonidin and (**c**,**d**) pelargonidin-3-O-glucoside with HAD.

Predictions of bio molecular interaction of small ligands such as flavonoids are important for prediction of their potential side effects. Swissadme is an online database that provides large set of data on structural properties of proteins and their interactions. Homosapien class of targets were chosen and the binding probability for pelargonidin and pelargonidin-3-O-glucoside were analyzed and depicted in Figure 4a–d [24,25,26]. The data sets received from the server revealed that pelargonidin highly prefers enzymatic receptor-based targets followed by transcription factor (TF). TF is a protein that controls the rate of transcription of genetic information from DNA to messenger RNA, whereas pelargonidin-3-O-glucoside prefers enzymatic targets and membrane receptors; hence it finds major applicability in cancer treatment. With reference to the target prediction report, enzyme-based targets are of common interest for both the compounds. Here, hydroxyacyl-coenzyme A dehydrogenase (HAD) PDB ID: 1f0y UniProt Name: HCDH_HUMAN was used as an enzyme target. This enzyme catalyzes the β-oxidation of fatty acids. In order to predict the suitable binding sites, HAD was docked with pelargonidin and pelargonidin-3-O-glucoside [30,31]. The docking mode with the lowest binding free energy between pelargonidin, pelargonidin-3-O-glucoside, and HAD is shown in Figure 5a,b. For HAD-pelargonidin bonding interactions and hydrogen bonding with Ala, Asn, and Gly residues were witnessed. Between HAD and pelargonidin-3-O-glucoside, two H-bond interactions with Asn and a hydrogen with Ala were witnessed. These results showed that the hydrogen bonds involved here are in association with HAD and continue to maintain the structural stability of these complexes within the binding pocket at site [32,33,34,35,36,37]. In addition, the binding free energy ΔG calculated by molecular docking was −35.23 kcal/mol (HAD-pelargonidin) and −18.41 kcal/mol (HAD-pelargonidin 3-O-glucoside). These data revealed the possible binding capability of two anthocyanins with HAD. 

### 3.5. Molecular Dynamics Analysis

**Figure 6:**
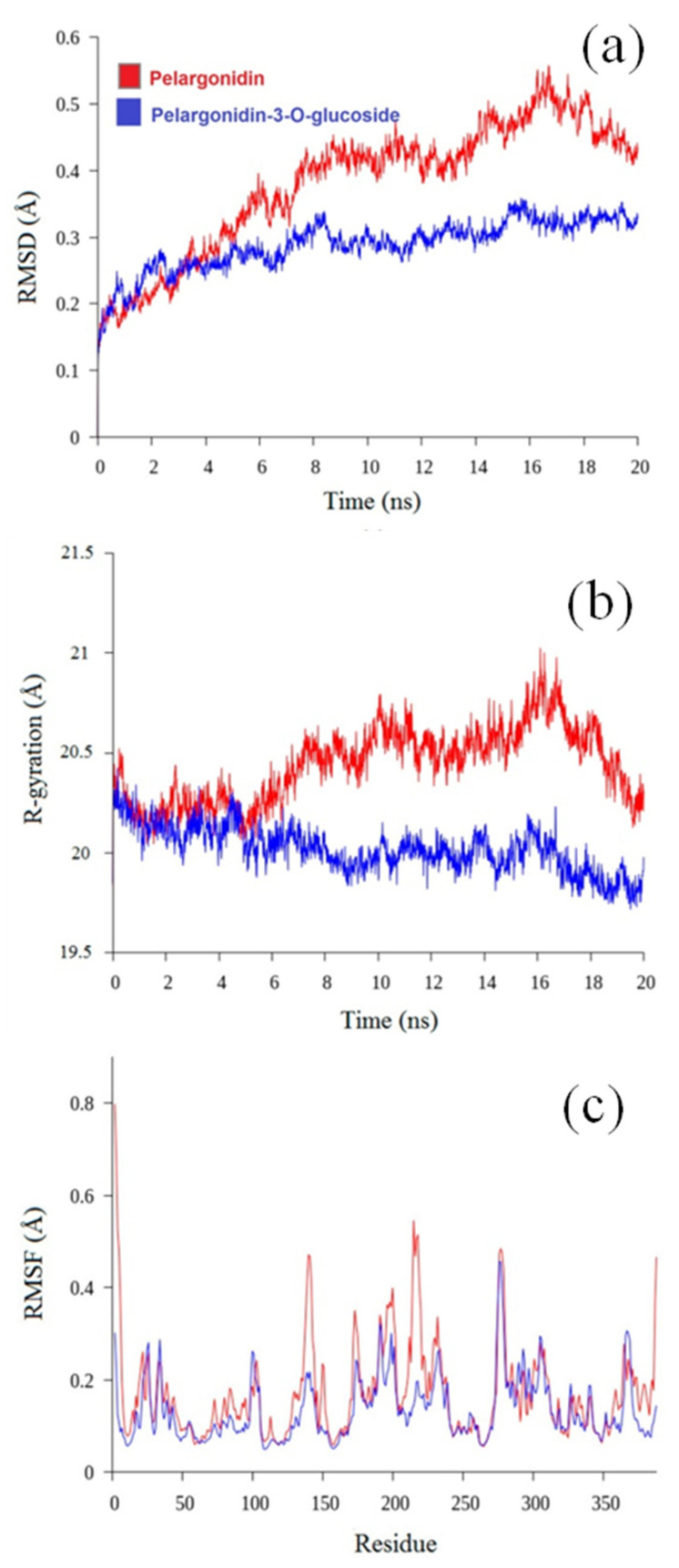
RMSD (**a**), radius of gyration (**b**) and RMSF (**c**) for pelargonidin and pelargonidin-3-O-glucoside with HAD.

The interactions between pelargonidin and pelargonidin-3-O-glucoside have been examined using the molecular docking method. To compare the differences between these compounds at the molecular level, a 20 ns MD simulation approach was employed. Using the GROMACS program, the stability and degree of interactions between HAD and pelargonidin and pelargonidin-3-O-glucoside were further explained. The trajectory data from the 20 ns MD simulations were then analyzed in order to determine the dynamic parameters of the two compounds. In the molecular dynamics research, each system was assessed using the root mean square deviation (RMSD), radius of gyration (Rg), solvent accessible surface area (SASA), and root mean square fluctuation (RMSF). The obtained results are displayed in Figure 6a–c.

#### 3.5.1. Root Mean Square Deviation 

RMSD is used to assess if the complex system has attained a stable state by calculating the average deviation between the complex’s current conformation and its actual conformation at a particular time. The RMSD value of the pelargonidin-HAD complex configuration remained rising throughout the simulation, as shown in Figure 6a, until it stabilized at around 4 Å after 20 ns. The pelargonidin-3-O-glucoside/HAD complex experienced only minor oscillations between 15 and 20 ns, but it quickly attained an RMSD value of about 38 Å beyond that time. These findings suggested that pelargonidin-3-O-glucoside had attained a stable state.

#### 3.5.2. Radius of Gyration

The time evolution of the radius of gyration (Rg) is useful for evaluating the kinetics of protein collapse. In order to verify the protein compactness, the radius of gyration of pelargonidin and pelargonidin-3-O-glucoside was quantified. Furthermore, the relationship between the radius of gyration and the simulation time was plotted (Figure 6b). The Rg of all the systems stabilized at about 20 ns, indicating that the MD simulation attained equilibrium 20 ns. Pelargonidin-HAD’s Rg values fluctuated between 2 and 2.5 nm throughout the simulation, demonstrating that the binding area had no impact on their structural characteristics. The pelargonidin-3-O-glucoside-HAD complex’s Rg value decreases during the course of the simulation (20 ns), and its average Rg value of 20 ± 0.02 Å reveals that their structure has become more compact. Thus, pelargonidin 3-O-glucoside-HAD complex’s gyration radius is less than that pelargonidin, and its structural tightness is superior to that of its neighbor.

#### 3.5.3. Root Mean Square Fluctuation (RMSF)

Figure 6c displays the time-averaged root mean square fluctuation (RMSF) values of HAD residues in the absence and presence of ligands plotted against residue numbers along the simulation trajectory, in order to evaluate the mobility of local proteins. The fact that the neat HAD’s RMSF values were often greater than those of the compound suggests that the HAD’s fluctuations were constrained by the ligands’ binding. The findings demonstrate that residues located far from the ligand-binding site are responsible for fluctuations higher than 0.4 Å. Additionally, the RMSF value is minimal and the most stable residue is the one in contact with the ligand.

As shown in Figure 6c, the RMSF of the ligand atom sites was also determined to examine any conformational changes. The outcomes demonstrate that there was little variability (<0.24 Å) in the compounds. Therefore, it can be said that during the simulation, the interactions between ligands were stable.

## 4. Conclusions

In the present investigation, the naturally occurring flavonoids pelargonidin and pelargonidin-3-O-glucoside were theoretically studied for their structural activity through various analyses such as frontier molecular orbital analysis, molecular electrostatic potential analysis, and molecular descriptive parameter. The obtained results are as follows. The initial step of this investigation was geometry optimization. With the help of density functional theory, using exchange correlation functional B3LYP and basis set 6-311G (d,p), ground state energies for the two flavonoids were obtained. Geometry corresponding to ground state minima was also simulated. Frontier molecular orbital analysis nominates pelargonidin to be more favorable for the radical scavenging process as it possesses the majority of electron localization over the B-ring and A-ring where –OH units are present. The energy gap for pelargonidin is 1.85 eV and for pelargonidin-3-O-glucoside it is about 1.91 eV, which again makes pelargonidin a nominal antioxidant. The electropositive region in the MEP surface of these compounds confirmed their responsivity towards the free radical scavenging action. The glucosyl unit present in pelargonidin-3-O-glucoside seems to be highly delocalized and suppresses the activity for its host. Molecular descriptive parameter values clearly stated that two flavonoids are better electron donors rather than electron acceptors. 

BOILED-egg plot analysis suggests that pelargonidin can be absorbed by intestine whereas pelargonidin-3-O-glucoised does not choose any absorption pathway. Target prediction reports showed that the flavonoid pelargonidin prefers enzymatic receptor-based targets followed by a protein that controls the rate of transcription of genetic information from DNA to messenger RNA, whereas pelargonidin-3-O-glucoside prefers enzymatic targets and membrane receptor; hence it finds major applicability in cancer treatment. By keeping the enzymatic target as common interest for both the compounds, molecular docking and MD simulations were performed with HAD and it was found that pelargonidin binds with the target effectively, thus favors inhibition easier. On the basis of stability data attained from MD simulations, pelargonidin-3-O-glucoside seems to be more stable than pelargonidin. Overall, these findings suggest that the structural activity and HAD target inhibition potency of pelargonidin was better than its derivative pelargonidin-3-O-glucoside.

## Figures and Tables

**Table 1:** Molecular descriptive parameter values of pelargonidin and pelargonidin-3-O-glucoside.

Molecular Descriptors (eV)	Eo (eV) of Pelargonidin	Eo (eV) of Pelargonidin-3–O–Glucoside
IP (eV)	4.07	4.19
EA (eV)	2.15	2.32
ω (eV)	0.95	0.93
S (eV)	0.52	0.53
Χ (eV)	3.11	3.25
η (eV)	5.07	5.68

## Data Availability

Upon reasonable request, the data supporting this investigation are available from the corresponding authors.

## Sample Availability

Not available.
